# The effects of transfusion load on renal function in intraoperative salvage autotransfusion: a retrospective cohort study for 200 patients

**DOI:** 10.3389/fphys.2025.1640701

**Published:** 2025-09-11

**Authors:** Honglan Wang, Meixin Zhang, Yu Gu, Weixin Zeng, Mei You, Wenxing Chen, Zhongshu Wu, Yahua Ye, Weitao Yu, Shuxuan Wang, Jingyang Zeng

**Affiliations:** ^1^ Department of Dermatology, Quanzhou First Hospital Affiliated to Fujian Medical University, Quanzhou, Fujian, China; ^2^ Department of Anesthesiology, Quanzhou First Hospital Affiliated to Fujian Medical University, Quanzhou, Fujian, China; ^3^ Department of Anesthesiology, The Fourth People’s Hospital of Zigong, Zigong, Sichuan, China; ^4^ Graduate School of Fujian Medical University, Fuzhou, Fujian, China

**Keywords:** acute kidney injury, creatinine, free hemoglobin, intraoperative salvage auto-transfusion, clinical investigation

## Abstract

**Objectives:**

This study aimed to investigate the effect of transfusion load on renal function during intraoperative salvage auto-transfusion.

**Methods:**

A total of 200 patients were assigned to five groups based on the transfusion load: 0–200 mL (Group A, n = 40), 201–400 mL (Group B, n = 40), 401–600 mL (Group C, n = 40), 601–800 mL (Group D, n = 40), >800 mL (Group E, n = 40). Serum creatinine (sCr) and free hemoglobin (FHb) concentrations were measured at preoperative baseline (T_0_), 1 h (T_3_), 2 h (T_4_), 12 h (T_5_), and 24 h (T_6_) post-auto-transfusion. FHb and sCr levels were also assessed in the blood storage tank before washed (T_1_) and in the reinfusion bag after washing (T_2_).

**Results:**

In Groups A, B, and C, there was no significant change in sCr levels between T0 and subsequent time points (T_3_, T_4_, T_5_, T_6_). However, in Groups D and E, sCr levels increased by more than 26.5 μmol/L from baseline at T_3_ and T_4_, with sustained elevations at T_3_, T_4_, and T_5_ compared to T0. FHb concentrations were higher in both T_1_ and T_2_ compared to T_0_, following a similar trend as sCr. Patients receiving more than 600 mL of autologous transfusion showed a transient increase in sCr at 1 and 2 h post-transfusion, consistent with acute kidney injury (AKI), which resolved by 24 h after transfusion.

**Conclusion:**

Attention should be paid to renal function in patients receiving large volumes (>600 mL) of salvaged blood during intraoperative auto-transfusion, as these patients may experience transient AKI, which resolves over time.

## Graphical Abstract

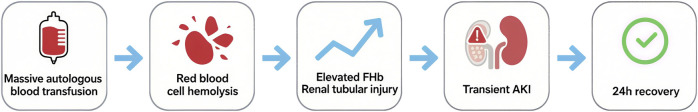

## Introduction

Intraoperative autologous blood transfusion is a common and convenient technique used to reinfuse scavenged blood back into the patient (Sikorski et al., 2017). Autologous blood transfusion is frequently employed during major surgical procedures where there is a significant risk of intraoperative blood loss (Yu et al., 2013). It is reported that 30%–50% of patients require transfusion in major orthopedic surgeries, among which autologous transfusion can meet 20%–40% of transfusion needs, particularly in spinal and pelvic surgeries (Barrie et al., 2022). This technique can help minimize the need for allogeneic blood transfusions, thereby reducing the risk of hemolytic and non-hemolytic adverse events (Flom-Halvorsen et al., 1999; Grainger and Catling, 2018). However, the increasing use of salvaged blood raises concerns about potential complications, including dilutional coagulopathy, bacterial contamination, and nonimmune hemolysis (Sikorski et al., 2017). While these issues have been well-studied, less attention has been given to the impact of autotransfusion on kidney function. The pathogenesis of autotransfusion-related AKI involves a dynamic interplay of systemic and renal-specific insults. Central to this process is free hemoglobin (FHb) overload. Patient baseline conditions also modulate acute kidney injury (AKI) susceptibility. Advanced age reduces renal reserve, with each 10-year increase raising AKI risk by 34% in aortic dissection surgery. Obesity amplifies systemic inflammation, increasing AKI risk by 23% per 5 kg/m2 increment. Moreover, surgical factors, particularly prolonged cardiopulmonary bypass (CPB), are major determinants of AKI risk (Kurland et al., 2024).

Given the case of a patient who developed AKI following autologous blood transfusion, confirmed by kidney biopsy (Minkara et al., 2017), it is important to further investigate the relationship between kidney function and autologous blood transfusion.

This study analyzed the changes in serum creatinine (sCr) and FHb levels in patients undergoing autotransfusion to assess the impact of transfusion volume on renal function. The aim of this study is to provide a clinical basis for the safe application of autologous blood transfusion.

## Methods

### Study design and subjects

From September 2018 to December 2022, a total of 200 patients who underwent orthopedic surgeries (including thoracic decompression and internal fixation, open reduction and plate internal fixation of pelvic fractures, lumbar decompression and spinal fusion, posterior fixation of lumbar pedicle screws, etc.) were selected for autotransfusion at our hospital. This study was a retrospective cohort study and was approved by the Institutional Review Board of Quanzhou First Hospital Affiliated to Fujian Medical University (#[2023]K093). Informed written consent was obtained from all participants. The study adheres to STROBE guidelines. Eligible patients were included based on their surgery dates, and each group concluded once the number of eligible participants reached 40.

#### Inclusion criteria

1. ASA classification II-III, age over 18 years;
2. Patients requiring autologous blood transfusion due to orthopedic surgery;
3. Meeting the indications for autologous blood transfusion treatment;
4. Patients with normal renal function, defined as those with sCr within the normal range based on the reference interval cited from the Chinese Health Industry Standard WS/T 404.5-2015.
5. Informed consent obtained from the patient and their legal guardian/family member.

#### Exclusion criteria

1. Patients who received transfusion treatment within the past 1 month;
2. History of urinary surgery, trauma, infection, or other urinary diseases, such as kidney tumor or drug-induced kidney injury;
3. Liver or kidney dysfunction, with alanine aminotransferase levels exceeding 2 times the normal upper limit, serum creatinine exceeding 2 times the normal upper limit, urine protein ≥ (++), to avoid the influence of severe renal insufficiency (CKD stage 3 and above, determined by the eGFR values less than 30 mL/min/1.73 m^2^) on study outcomes;
4. Contraindications for autologous blood transfusion, including surgical field contamination, malignant tumors, or wound infection;
5. Any other clinical conditions deemed inappropriate for inclusion by the anesthesiologists considered;
6. Patients with a solitary kidney.
7. Patients with anemic preoperatively.
8. Patients with an additional transfusion.
9. Patients with major postoperative events occurred (severe complications within 24 h postoperatively that may interfere with renal function assessment, such as massive hemorrhage requiring reoperation for hemostasis, septic shock, cardiovascular/cerebrovascular accidents and multiple organ failure).

### Groups

According to the volume of intraoperative reinfused salvaged blood, patients were divided into five groups, each containing 40 cases (n = 40) as follows: Group A: 0–200 mL; Group B: 201–400 mL; Group C: 401–600 mL; Group D: 601–800 mL; Group E: above 800 mL.

### Operation

No preoperative medication was administered. Upon admission, oxygen inhalation was initiated, and various hemodynamic parameters were monitored. Intravenous injections of propofol (2–3 mg/kg), fentanyl (3–5 μg/kg), and rocuronium (0.6 mg/kg) were given, followed by mechanical ventilation after endotracheal intubation. Sevoflurane, remifentanil, and rocuronium were used to maintain anesthesia throughout the procedure. Basic blood pressure was maintained at 80% of baseline values during the operation. Medication and fluid infusions were adjusted based on hemodynamic monitoring and the surgical process. No allogeneic blood transfusion was administered during the surgery, and no autologous blood transfusion was performed postoperatively.

### Implementation of autologous blood transfusion

The C.A.T.S. Plus Continuous Autologous Blood Recovery Machine (Fresenius Kabi Company) was set up, and saline containing heparin (25,000 IU) was prepared according to the operating protocol prior to surgery. Using the lumen suction tubing, fluid was collected from the operative field. The collected fluid was filtered into a sterile blood storage tank. When the fluid volume in the storage tank reached 600–1,000 mL, the washing phase began. The volume of saline used for washing was three times the centrifuged blood volume, with a hematocrit of approximately 40%–60%. At a rate of 175 mL/min, the fluid was washed five times, then transferred to the reinfusion bag. The washed fluids were subsequently reinfused into the patient under careful monitoring.

### Collection of the tested specimen

After mixing the blood in the three-way piston twice (Alberts et al., 2017), 6 mL of test specimens were drawn from patients either from the blood storage tank connection end or the blood transfusion bag access end. The following time points were established for blood collection:

T_0_: blood was collected from patients before surgery;

T_1_: blood was collected from the blood storage tank before washing;

T_2_: blood was collected from the reinfusion bag after washing;

T_3_: blood was collected from patients at 1 h after autotransfusion;

T_4_: blood was collected from patients at 2 h after autotransfusion;

T_5_: blood was collected from patients at 12 h after autotransfusion;

T_6_: blood was collected from patients at 24 h after autotransfusion.

### Determination of sCr

Patients’ blood was collected at different time points (before surgery, 1 h, 2 h, 12 h, or 24 h after autotransfusion) for biochemical examination. Serum levels of sCr were determined using the enzymatic method with the Human Creatinine Assay Kit (#80189, Crystal Chem), following the manufacturer’s protocol. The sCr levels were recorded and analyzed as one of the primary indicators of kidney function. According to the AKIN standard, an increase in sCr of more than 26.5 μmol/L from baseline is considered as AKI (Levey et al., 2020).

### Determination of the concentration of FHb (Trinder method)

The FHb concentration was measured using the FHb kit (Ruierda Biological Technology, Beijing, batch number: 181204), with analysis performed by a spectrophotometer blood gas analyzer, following the manufacturer’s instructions.

### Statistical analysis

Based on pilot data showing about 30% AKI incidence in high-transfusion groups (>600 mL), with α = 0.05 and β = 0.2, PASS 15 software calculated a minimum of 38 cases per group. No missing data were observed in this study and there were 40 cases were included to ensure power in this study. Data were analyzed using IBM SPSS Statistics 20.0 software (Chicago, IL, United States). Normality was tested via Shapiro-Wilk test and Q-Q plot visualization. sCr and FHb data were normally distributed (P > 0.05). Continuous variables are expressed as the mean ± standard error (SE) and analyzed using Student's t-test. A two-way repeated measures analysis was conducted to assess the differences in FHb and sCr concentrations at different time points. Categorical variables are presented as counts and proportions and were analyzed using the Chi-square or Fisher’s exact test. A p-value of <0.05 was considered statistically significant.

## Results

### Basic clinical characteristics

Demographic and baseline clinical data were comparable across the five groups (all *P* > 0.05, Table 1). There were no significant differences in sex distribution, ASA classification (II/III), age, or body mass index (BMI) between groups. The prevalence of diabetes mellitus (12.5%–17.5%) and hypertension (25%–32.5%) was similar across groups, confirming balanced baseline comorbidity profiles.

**TABLE 1:** Demographic characteristics of the patients (n = 40).

Group	Men	Women	ASA		Age	BMI (kg/m^2^)	Diabetes mellitus		Hypertension	
			Classification (cases)							
			II	III			Yes	No	Yes	No
Group A	22 (55%)	18 (45%)	33 (82.5%)	7 (17.5%)	59.1 ± 12.1	22.4 ± 1.7	6 (15%)	34 (85%)	13 (32.5%)	27 (67.5%)
Group B	19 (47.5%)	21 (52.5%)	33 (82.5%)	7 (17.5%)	57.3 ± 14.0	22.3 ± 1.8	7 (17.5%)	33 (82.5%)	10 (25%)	30 (75%)
Group C	18 (45%)	22 (55%)	39 (97.5%)	1 (2.5%)	61.2 ± 12.4	22.1 ± 1.7	5 (12.5%)	35 (87.5%)	12 (30%)	28 (70%)
Group D	21 (52.5%)	19 (47.5%)	34 (85%)	6 (15%)	60.5 ± 14.2	22.6 ± 1.7	6 (15%)	34 (85%)	11 (27.5%)	29 (72.5%)
Group E	22 (55%)	18 (45%)	35 (87.5%)	5 (12.5%)	58.9 ± 11.8	22.7 ± 1.7	7 (17.5%)	33 (82.5%)	12 (30%)	28 (70%)
χ^2^/F	1.321		5.482		0.56	0.69	1.842		4.621	
P	0.858		0.241		0.691	0.59	0.991		0.195	

Values are expressed as n (%).

Data are presented as mean ± standard error or n (%).

### sCr and FHb dynamics in response to transfusion

**FIGURE 1:**
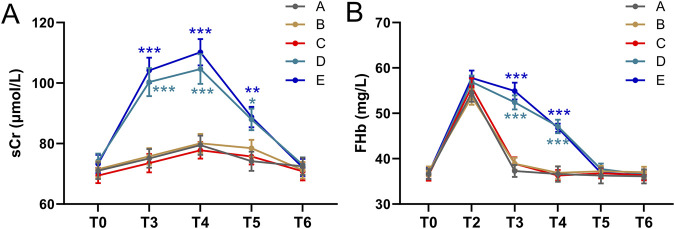
Comparisons of sCr **(A)** and FHb **(B)** among five groups of patients before and after autologous blood transfusion ((T0, T3, T4, T5, T6) and (T0, T2, T3, T4, T5, T6)). Data were shown with mean ± SEM. ***p < 0.001 means the difference of at this time point compared to the timepoint of T0. Two-way ANOVA following Tukey’s multiple comparisons test.

There was no significant difference in sCr levels between T_3_, T_4_, T_5_, T_6_, and T_0_ in groups A, B, and C (all p > 0.05, Figure 1A). In groups D and E, sCr levels at T_3_, T_4_, and T_5_ were significantly higher than at T_0_, but no significant difference was observed between the sCr levels at T_6_ and T_0_ (Figure 1A). In groups D and E, sCr increased significantly compared to baseline at T_3_ and T_4_ (Figure 1A). Which returned to normal by 24 h.

No abnormal values were identified using the method of studentized residuals (i.e., values greater than 3 standard deviations). The FHb concentration in the blood from the reinfusion bag after washing (T_2_) was higher than that in the patients’ blood. When comparing the FHb concentration in the patients’ blood, a statistically significant difference was found among the five groups (F = 4597.219, p < 0.001). Additionally, the difference between different time points was also statistically significant (F = 584.815, p < 0.001) (Figure 1B). At T_3_ and T_4_, the FHb concentration in groups D and E was significantly higher than in groups A, B, and C (*P* < 0.001, Figure 1B). In groups D and E, the FHb concentration at T_3_ and T_4_ was significantly higher than at T_0_, T_5_, and T_6_. These results suggest that FHb concentration in groups D and E increased significantly at T_3_ and T_4_, then returned to baseline at T_5_ and T_6_. The trend in FHb concentration in patients’ blood closely mirrored the trend in sCr levels.

### sCr differences between groups

**FIGURE 2:**
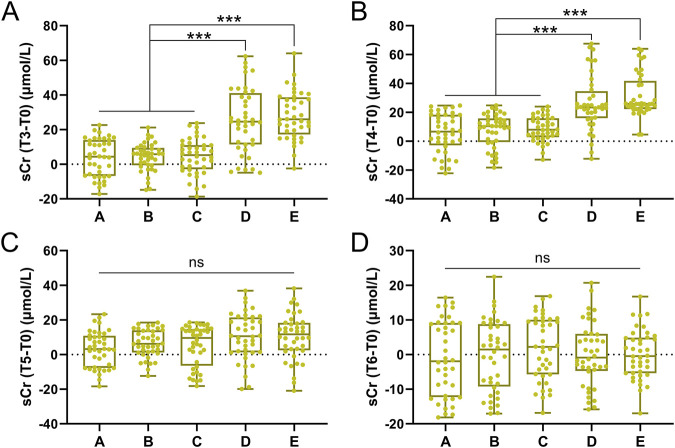
Comparisons of sCr difference of T3-T0 **(A)**, T4-T0 **(B)**, T5-T0 **(C)**, T6-T0 **(D)** among five groups. Data was shown with Box plot. ***p < 0.001 from Brown-Forsythe ANOVA test following with Dunnett’s T3 multiple comparisons test.

The magnitude of sCr elevation (ΔsCr = Tx-T0) differed significantly between groups. At T3 and T4, ΔsCr in Groups D and E was substantially higher than in Groups A–C (p < 0.001, Figures 2A,B). By T5, ΔsCr in high-transfusion groups decreased but remained slightly elevated, while no significant changes were observed in low-transfusion groups (Figure 2C). By T6, ΔsCr returned to near baseline in all groups (Figure 2D).

### Analysis of AKI incidence

**FIGURE 3:**
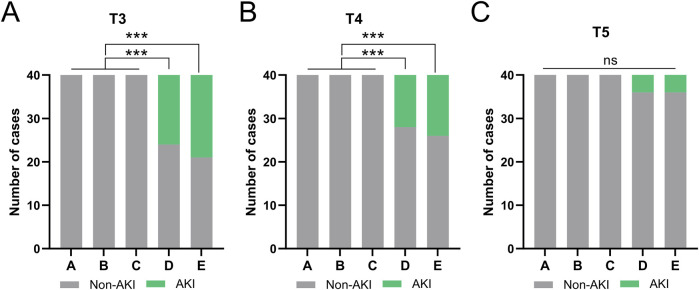
Comparisons of the occurrence AKI at the time of T3 **(A)**, T4 **(B)**, T5 **(C)** among five groups. ***p < 0.001 from Fisher’s exact test.

AKI was defined using the AKIN criteria (≥26.5 μmol/L increase in sCr within 48 h). Results showed no AKI cases in the low-transfusion groups (A, B, C) at all time points. The incidence of AKI in the high-transfusion groups (D, E) changed dynamically over time (Figures 3A–C). At T3 (1 h post-transfusion), there were 16 cases (40%) in Group D and 19 cases (47.5%) in Group E met the AKI criteria. At T4 (2 h post-transfusion), there were 12 cases (30%) in Group D and 14 cases (35%) in Group E still met the AKI criteria. At T5 (12 h post-transfusion), AKI cases decreased significantly, with 4 cases (10%) in Group D and 4 cases (10%) in Group E. There were no significant differences compared to low-transfusion groups (A, B, C) groups.

All AKI cases received enhanced fluid management and renal function monitoring without renal replacement therapy; sCr returned to baseline within 24 h (no difference between T6 and T0).

### Correlation between sCr and FHb

**FIGURE 4:**
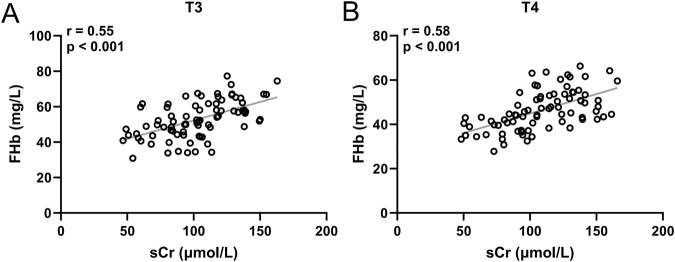
Pearson correlation analysis of sCr and FHb in group D and E at the time of T3 **(A)** and T4 **(B)**. 80 patients at each timepoint.

To verify the correlation between the increase in sCr and FHb, we analyzed the correlation between sCr levels and FHb levels in patients from the High-transfusion groups (D, E) at both T3 (Figure 4A) and T4 time points (Figure 4B). The results demonstrated that in patients from Groups D and E, there was a significant positive correlation between sCr levels and FHb levels at both T3 and T4 time points, with correlation coefficients r = 0.55 and 0.58, respectively, and p < 0.001.

### Risk factors for AKI during intraoperative salvage auto-transfusion

Multivariate logistic regression model was used to analyze the risk factors for AKI during intraoperative salvage auto-transfusion. Age, BMI, ASA classification and transfusion load were set as covariates, and AKI occurrence as the outcome. Multivariate logistic analysis indicated that age (odds ratio = 1.471 (1.084–2.413), P = 0.012), body mass index (odds ratio = 1.782 (1.146–3.325), P = 0.009) and high-transfusion (odds ratio = 2.015 (1.225–3.796), P = 0.002) were risk factors for impaired renal function.

## Discussion

In this study, data from 200 patients who received different volumes of autologous transfusion of intraoperative salvaged blood were retrospectively reviewed and analyzed. sCr levels were assessed according to the AKIN standard. For patients receiving less than 600 mL of autologous blood transfusion, no signs of AKI were found within 24 h after autotransfusion. However, in patients receiving more than 600 mL of autologous blood transfusion, sCr levels indicated the presence of AKI at 1 and 2 h after autotransfusion, with levels returning to normal by 24 h after transfusion.

The successful use of autologous transfusion in our study supports the idea that intraoperative salvaged blood is a safe technique for patients undergoing major surgeries, as indicated in previous studies (Goulet et al., 1989; Chanda et al., 2002). Autologous transfusion has been reported as an effective and safe method for reducing the need for allogeneic blood transfusion in various surgical procedures. Transfusion of allogeneic blood is associated with an increased risk of hepatitis, AIDS, hemolysis, transfusion coagulopathy, and allergic reactions (Carson et al., 2017). Autologous transfusion can reduce the absolute risk of allogeneic transfusion by 23% (Carless et al., 2010).

The increase in sCr in patients with autologous transfusion volumes greater than 600 mL suggests that intraoperative blood salvage autotransfusion should be closely monitored due to its potential risk of several complications. Hematuria has been reported in 3 cases during the use of low-volume salvage autotransfusion (Keverline and Sanders, 1998), which indicates the possibility of kidney injury during salvage autotransfusion. A case study reported a patient with AKI demonstrated by renal biopsy (Minkara et al., 2017). It was found that the autologous blood transfusion volume was correlated with renal dysfunction in the open repair of suprarenal aortic aneurysms (Godier et al., 2012). Accurate markers of AKI during autotransfusion are difficult to utilize. The primary estimate of AKI in our study was based on the postoperative creatinine serum level variation, as compared with the baseline preoperative serum creatinine level. The results of sCr in this study seem to indicate that autologous transfusion volumes less than 600 mL exerted no or an acceptable influence on kidney function. However, for patients who received autologous transfusion volumes greater than 600 mL, deliberate consideration for kidney protection is prudent.

With the recovery of autologous blood during the operation, the senescent and degenerated RBCs are prone to rupture and hemolysis during high-speed centrifugation and washing. FHb are released from hemolyzed RBCs and have been addressed as an etiology of AKI (Kerchberger and Ware, 2020). Our study found that the concentration of FHb in the autologous blood recovery machine was greatly reduced after washing. In patients with autologous blood volumes greater than 600 mL, FHb increased significantly at 1 h and 2 h after autotransfusion. The greater the autologous blood volume, the more FHb may enter the patients’ circulation, potentially negatively impacting kidney function. In this study, there were two cases of dark-colored urine in patients with salvaged blood volumes exceeding 800 mL (group E), but after 24 h, the urine color returned to normal, indicating that the kidney injury is reversible and transitory. Many factors, such as renal ischemia and systemic inflammatory activation, have been demonstrated to elicit the development of AKI (Lieberthal and Nigam, 2000). The outcomes in our study seem to show the independent risk of FHb on kidney function during intraoperative blood salvage autotransfusion (Zeng et al., 2021). An efficient strategy for reducing the production and rapidly scavenging FHb should be considered when the salvaged blood volume exceeds 600 mL.

The implications of AKI in patients undergoing orthopedic surgeries are significant, as the condition can lead to prolonged hospital stays, increased morbidity and mortality, and long-term kidney dysfunction. By enhancing our understanding of how renal function is affected in high-risk surgical contexts, we can develop more effective prevention and management strategies to protect renal health. These strategies should involve a comprehensive approach that includes preoperative risk assessment, intraoperative hemodynamic management, and postoperative monitoring. Ultimately, improving the care of these patients will contribute to better outcomes, reduced complications, and a higher quality of life post-surgery.

A review of anesthesia records showed that hemodynamics remained stable within ±20% of preoperative values in all cases. This stability was maintained through timely fluid resuscitation and vasoactive drug use. In patients with significant blood loss, hemodynamic stability was achieved by reinfusing autologous blood along with fluid resuscitation and vasoactive drugs. Despite individual variations, the mean arterial pressure remained within ±20% of preoperative levels, with no episodes of hypertension or hypotension. This rules out blood loss-induced hypotension as a cause of kidney injury, allowing us to exclude its influence on our study results.

Potential biases in preoperative severity assessment primarily relate to ASA classification and baseline renal reserve. This study controlled for interference from extremely severe patients through strict inclusion criteria (ASA II-III), with balanced distribution of ASA classes across groups, minimizing confounding from uneven disease severity. Additionally, all patients had preoperatively verified normal renal function via sCr according to the Chinese Health Industry Standard WS/T 404.5-2015, avoiding interference from baseline renal function differences in AKI diagnosis.

The pathological mechanism of autotransfusion-related AKI differs significantly from other perioperative AKI subtypes. Previous renal biopsy studies have shown that autologous blood transfusion-associated AKI is characterized by renal tubular epithelial cell swelling, cast formation, and mild interstitial inflammation (Tong et al., 2021), with a core mechanism of FHb-mediated tubular toxicity: direct tubular luminal obstruction by FHb; oxidative stress in epithelial cells via Fenton reaction-derived free radicals; renal vasoconstriction due to depletion of endothelial nitric oxide (NO) (He et al., 2025). This contrasts with glomerular ischemic changes in ischemic AKI and microvascular inflammatory responses in septic AKI. The dynamic correlation between FHb and sCr in this study further supports this mechanism, while the transient nature of AKI (resolution within 24 h) aligns with the reversible characteristics of tubular injury, distinguishing it from persistent AKI caused by irreversible glomerular damage.

There are several limitations that should be noted in the current study. First, as a retrospective study with a small sample size of 200 participants, the findings have limited generalizability. Second, in the orthopedic surgeries included, most patients did not require more than 800 mL of salvaged blood infusion, which may limit the scope for further research in this area. Additionally, this study did not include a control group receiving allogeneic transfusion, precluding direct comparison of renal safety between autologous and allogeneic transfusion modalities. However, this was not the primary focus of the current work, which aimed to investigate dose-dependent effects of autologous transfusion itself. Third, serum FHb levels are expected to be lower in autologous blood transfusions than in allogeneic transfusions, but we did not compare the FHb levels between the two types of transfusions. Furthermore, due to the retrospective nature of the study, urine output records were incomplete in some cases and thus not included in the analysis. Blood urea nitrogen was not used as a primary indicator because it is significantly influenced by postoperative fluid resuscitation, which may limit the comprehensiveness of AKI assessment. Finally, this study focuses exclusively on orthopedic surgeries, which may limit the ability to apply the findings to other high-blood-loss surgical procedures. Future research could broaden its scope to include a wider variety of surgical interventions. However, our study highlights the relationship between salvaged blood volume and kidney function, providing primary research on kidney damage due to FHb. When considered alongside previous studies and our results, it suggests that AKI may develop secondary to FHb when the volume of salvaged blood reinfused exceeds 600 mL.

## Conclusion

In summary, autologous blood transfusion of more than 600 mL in orthopedic surgery may lead to acute renal function damage. Therefore, close attention should be paid to kidney function during intraoperative salvaged blood transfusion.

## Data Availability

The original contributions presented in the study are included in the article/supplementary material, further inquiries can be directed to the corresponding author.
